# Spike-shape dependence of the spike-timing dependent synaptic plasticity in ferroelectric-tunnel-junction synapses

**DOI:** 10.1038/s41598-019-54215-w

**Published:** 2019-11-28

**Authors:** P. Stoliar, H. Yamada, Y. Toyosaki, A. Sawa

**Affiliations:** National Institute of Advanced Industrial Science and Technology (AIST), Tsukuba, Ibaraki 305-8565 Japan

**Keywords:** Applied physics, Electronic devices

## Abstract

Resistive switching (RS) devices have attracted increasing attention for artificial synapse applications in neural networks because of their nonvolatile and analogue resistance changes. Among the neural networks, a spiking neural network (SNN) based on spike-timing-dependent plasticity (STDP) is highly energy efficient. To implement STDP in resistive switching devices, several types of voltage spikes have been proposed to date, but there have been few reports on the relationship between the STDP characteristics and spike types. Here, we report the STDP characteristics implemented in ferroelectric tunnel junctions (FTJs) by several types of spikes. Based on simulated time evolutions of superimposed spikes and taking the nonlinear current-voltage (*I*-*V*) characteristics of FTJs into account, we propose equations for simulating the STDP curve parameters of a magnitude of the conductance change (Δ*G*_max_) and a time window (τ_C_) from the spike parameters of a peak amplitude (*V*_peak_) and time durations (*t*_p_ and *t*_d_) for three spike types: triangle-triangle, rectangular-triangle, and rectangular-rectangular. The power consumption experiments of the STDP revealed that the power consumption under the inactive-synapse condition (spike timing |Δ*t*| > τ_C_) was as large as 50–82% of that under the active-synapse condition (|Δ*t*| < τ_C_). This finding indicates that the power consumption under the inactive-synapse condition should be reduced to minimize the total power consumption of an SNN implemented by using FTJs as synapses.

## Introduction

**Figure 1:**
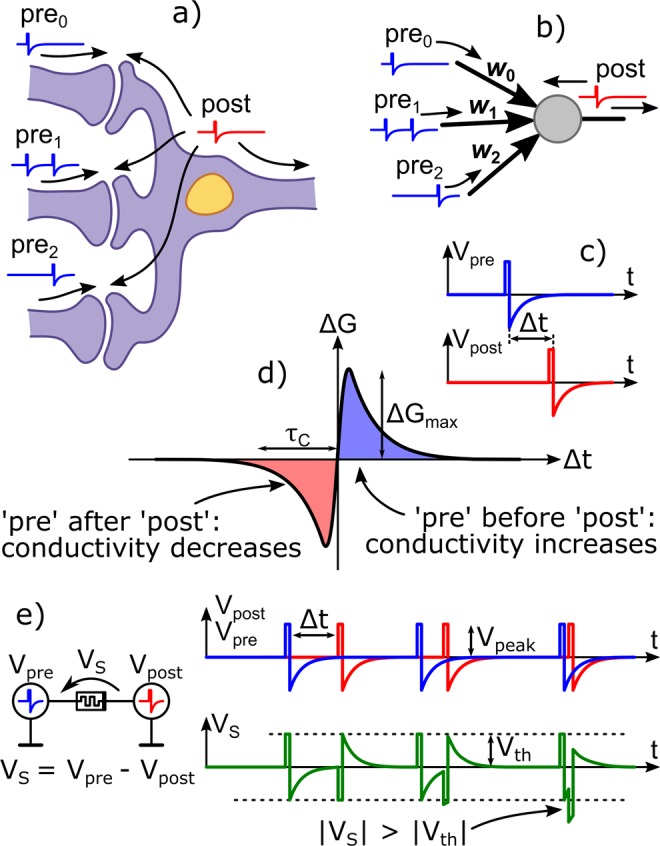
Neuromorphic system and STDP-based SNN. (**a**) Schematic image and (**b**) circuit model of a post neuron with three synapses. Pre_a_ (a = 1, 2, and 3) and post are pre- and post-spikes, and *w*_b_ (b = 1, 2, and 3) is the synaptic weight of the synapse. (**c**) Schematic images of pre- and post-spikes and (**d**) a typical STDP curve. Δ*t* is spike timing. (**e**) Model of the artificial synapse implemented by using the FTJ and time evolutions of pre- (blue), post- (red) spikes, and superimposed spikes (green). *V*_peak_ is the peak amplitude of the spike, *V*_s_ is the peak amplitude of the superimposed spikes, and *V*_th_ is the threshold voltage of resistive switching in the FTJ device.

In the internet of things era, energy-efficient computing systems are required to process the large amounts of data collected by sensors, such as in image and sound recognition. However, in conventional computing systems based on the von Neumann architecture, the frequent data transfer between spatially separated logic and memory units limits the processing speed, leading to energy inefficiency; this problem is known as the von Neumann bottleneck^1,2^. During the past decade, to overcome this problem, neural networks based on electronic devices have been intensively investigated^3–5^. In particular, spiking neural networks (SNNs) have been attracting increasing interest as a highly energy-efficient computing system. A typical SNN is composed of a tremendous set of computing nodes, which are regarded as artificial neurons^6^. Artificial neurons receive many inputs from other neurons in the form of electrical pulses or spikes. Artificial synapses weight input spikes and then summed up in a recipient neuron. After receiving the input spikes, the neuron performs a simple computation and generates output spikes^7^. This computation concept is taken from a biological system (Fig. 1a).

For SNNs, spike-timing-dependent plasticity (STDP) is one of the most studied learning mechanisms^8^. In the STDP mechanism, each artificial synapse receives *pre*-spikes and *post*-spikes from previous and post neurons, respectively (Fig. 1b). The weight of the synapse (synaptic weight) increases or decreases depending on the relative time difference (Δt) between pre- and post-spikes (Fig. 1c). Figure 1d presents a typical STDP curve in which a synaptic weight increases when a post neuron generates post-spikes immediately after receiving the pre-spikes (Δt > 0), meaning that the inputs are essential for the computation and are thus reinforced. On the other hand, when a post neuron receives pre-spikes after generating post-spikes (Δt < 0), the synaptic weight decreases. Resistive switching (RS) memories or memristors are often used to implement STDP artificial synapses in SNN circuits^9^ because they have the functions needed for an artificial synapse, such as nonvolatility, reversibility, and resistance (conductance), and can be continuously adjusted (Fig. 1e). Therefore, for SNNs composed of RS memories, the synaptic weight corresponds to the conductance of RS memories.

Several types of spike shapes have been proposed to date to implement STDP learning with RS memories^10^. The different shapes of spikes lead to the different shapes of STDP curves. Simplistically, we characterized the shape of the STDP curve by two parameters, i.e., the amplitude of the conductivity modulation (Δ*G*_max_) and the time window (τ_C_), as described in Fig. 1d. These two values depend not only on the resistive switching characteristics of the RS memories but also on the shape of the spikes. Moreover, these parameters influence the computation performance of the SNNs. The spike-shape and timing dependence of STDP curves has been studied in RS memories^11,12^. However, there have been few systematic investigations on the spike-shape dependence of STDP characteristics in RS memories, which are needed to establish design guidelines for STDP artificial synapses.

Here, we report on the spike-shape dependence of STDP characteristics in RS memories, i.e., artificial synapses. In this study, we employed ferroelectric tunnel junctions (FTJs) with a BaTiO_3_ (BTO) ferroelectric barrier as an RS memory^13^. FTJs are metal/ferroelectric insulator/metal junctions and show nonvolatile resistive switching in association with polarization reversal in the ferroelectric barrier, meaning that the resistive switching of FTJs is based on an electronic process^14,15^. Moreover, the resistance of FTJs can be changed continuously by tuning both the amplitude and the duration of the applied voltage pulses^16,17^. Because of this feature, STDP functionality has been demonstrated in FTJs^18–20^.

It should be noted that FTJs based on polarization reversal are expected to show much better switching stability than conventional RS memories based on conductive filaments, i.e., chemical reactions. Excellent switching stability is essential to ensure the reproducibility and reliability of results obtained from repetitive measurements, such as the STDP measurements. Thus, FTJs are considered suitable for our STDP experiment. To ensure the reproducibility of the results, we confirmed that our BTO-based FTJs showed no degradation of STDP characteristics after the completion of all measurements (see Supplementary Information for details). In this study, we demonstrated that the shape of the spikes influences the Δ*G*_max_ and τ_C_ values of the STDP curves in the BTO-based FTJs. The energy consumption by the update of synaptic weight dependent on the shape of spikes was also investigated. These results are discussed from the perspective of the relationship between the threshold voltage for resistive switching and the amplitude and the duration of superimposed spikes applied to the devices.

## Results

### Resistive switching characteristics

**Figure 2:**
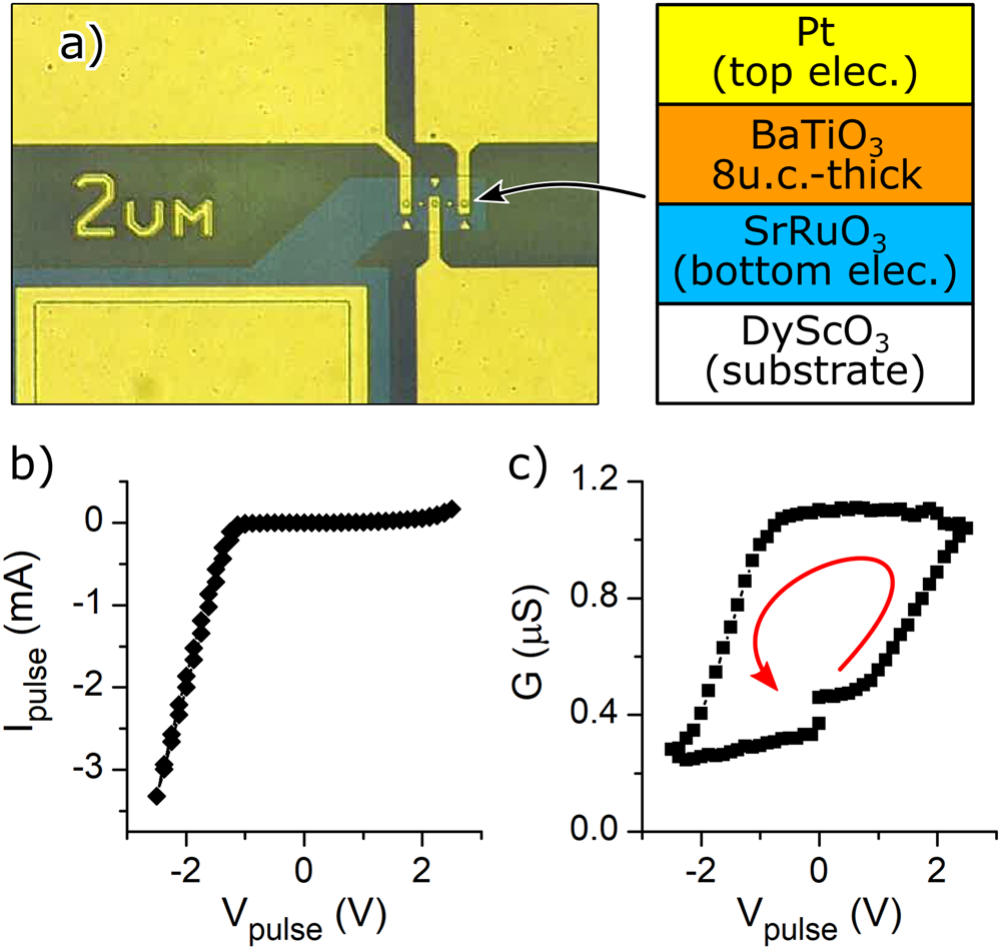
Image and electric properties of the BaTiO_3_-base FTJ. (**a**) Optical microscope image and cross-sectional schematic image of the BaTiO_3_-based FTJs. (**b**) Typical *I*-*V* curve and (**c**) typical hysteresis switching loop (HSL) of the FTJ measured by applying pulsed voltages.

The optical microscopy image and schematic of the BTO-based FTJs used in this study are shown in Fig. 2a. The junction area was 4 μm^2^, and the bottom and top metal electrodes were SrRuO_3_ and Pt, respectively. The details of sample preparation are described in the Methods section. Figure 2b presents the pulsed current-voltage (*I*-*V*) characteristics measured by applying a sequence of voltage pulses *V*_pulse_ (0 → 2.5 V → −2.5 V → 0, in 0.125 V increments, and a pulse duration of 3 ms). We observe an asymmetry (rectification) in the *I*-*V* characteristics resulting from an asymmetric potential distribution in the FTJ, as discussed in our previous report^13^. Note that in this work, we use the word “spike” to indicate complex voltage pulses utilized for the STDP measurements. On the other hand, we use the term “pulse” for a rectangular-shaped voltage pulse used for the standard electrical characterization of the FTJs.

To gain more insight into the memory properties of BTO-based FTJs, we also measured the hysteresis switching loop (HSL)^21^. Figure 2c shows the HSL of the BTO-based FTJ. Note that each data point in an HSL corresponds to a remnant conductance measured by applying a reading pulse (0.25 V) after a writing pulse (*V*_pulse_). We also confirmed from repeated measurements of HSLs that the BTO-based FTJ showed practically nonvolatile resistive switching (see Supplementary Information). These results indicate that the BTO-based FTJ meets the requirements for an artificial synapse: it shows practically nonvolatile and reversible resistive switching and that the conductance (resistance) can be continuously adjusted by setting the amplitude of the writing pulse to an appropriate value. We also confirm the presence of threshold voltages for resistive switching at approximately ± 1 V. Such threshold behaviour of resistive switching was often observed for FTJs, and the threshold voltages coincide with the coercive voltages of ferroelectric barriers^16,17^. In our FTJs, we have confirmed the coincidence of threshold voltage and the coercive voltage obtained by a piezo-response force microscopy, indicating that the ferroelectric polarization-reversal is a dominant cause of resistive switching^13^. This threshold behaviour is useful for implementing STDP, as will be discussed later.

Note that the conductance change of HSL in our FTJ was much smaller than that of the FTJs composed of Nb-doped SrTiO_3_ bottom-electrodes^22^. Although a large conductance change of FTJs is suitable for synaptic applications, we used prototypical Pt/BTO/SRO FTJs^23^ in this study to investigate basic STDP properties of FTJs.

### STDP characteristics

**Figure 3:**
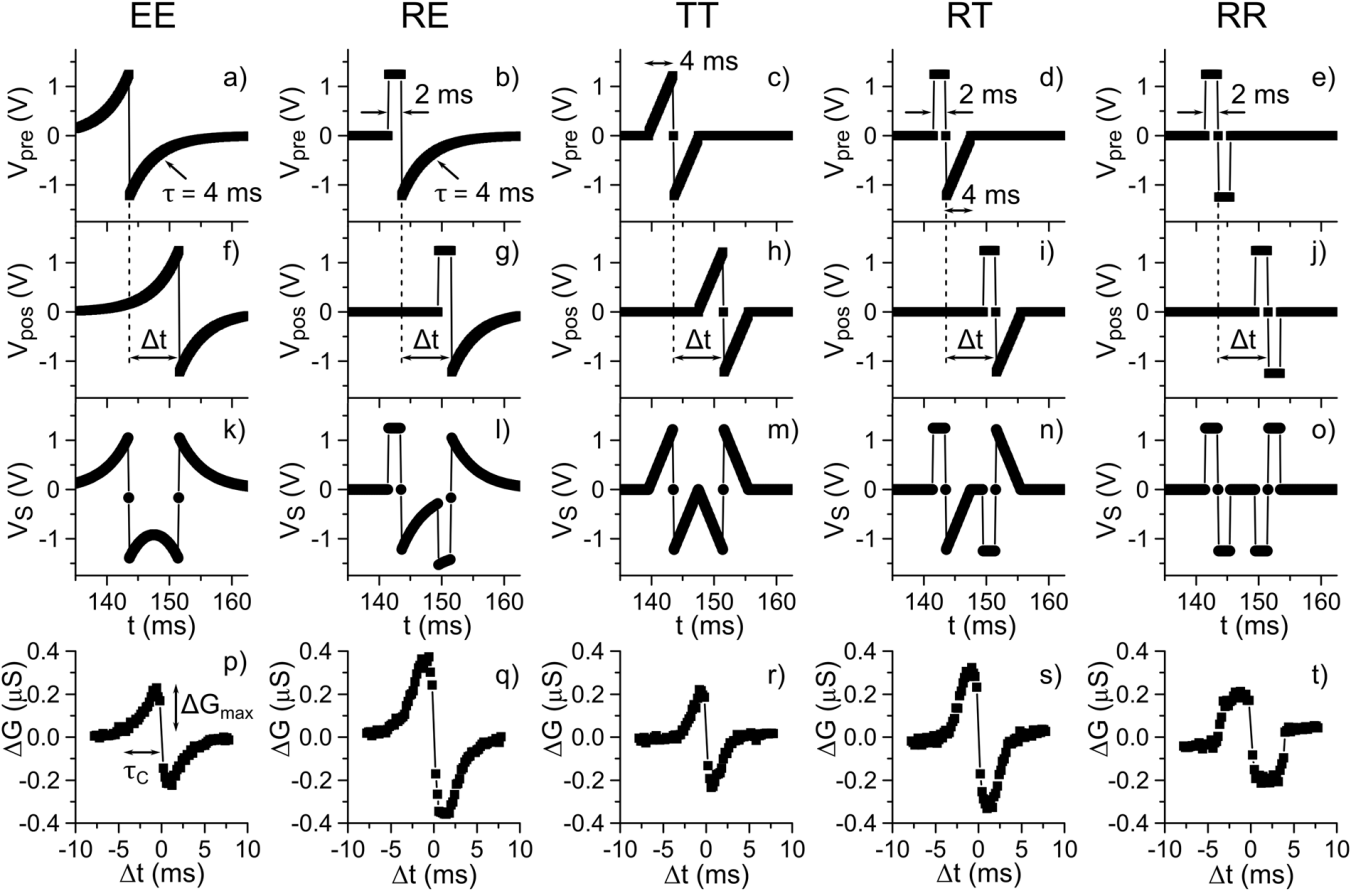
STDP curves for five different types of spikes. (**a**) Pre- and (**f**) post-spikes consisting of the exponential-decay pulses (EE-type spike) and (**k**) EE-type superimposed spike. (**b**) Pre- and (**g)** post-spikes consisting of the rectangular and exponential-decay pulses (RE-type spike), and (**l**) RE-type superimposed spike. (**c**) Pre- and (**h**) post-spikes consisting of triangle and triangle pulses (TT-type spike) and (**m**) TT-type superimposed spike. (**d**) Pre- and (**i**) post-spikes consisting of the rectangular and triangle pulses (RT-type spike) and (**n**) RT-type superimposed spike. (**e**) Pre- and (**j**) post-spikes consisting of the rectangular pulses (RR-type spike) and (**o)** RR-type superimposed spike. (**p**–**t**) STDP curves for EE-, RE-, TT-, RT-, and TT-type spikes, respectively. τ_C_ and Δ*G*_max_ are the time window and amplitude of the conductance change in the STDP curve.

To implement an STDP synapse using our FTJs, we may follow the standard procedure of STDP by applying a pre-spike *V*_pre_(t) to one terminal and a post-spike *V*_pos_(t) to the other (Fig. 1e)^9^. However, in this study, we adopted an equivalent procedure that is more convenient from the experimental point of view. One of the terminals was connected to ground, and the superimposed signal *V*_S_(*t*) [ = *V*_pre_(*t*) – *V*_pos_(*t*)] was applied to the other terminal. In this study, we investigated the STDP characteristics of our FTJs by applying five different types of superimposed signals (Fig. 3k–o) consisting the corresponding pre-spikes (Fig. 3a–e) and post-spikes (Fig. 3f–j). Here, we classify the types of spikes as follows. For the spike shown in Fig. 3a,f, because it consists of two pulses whose amplitude decays exponentially with time, this type is classified as the EE type. The spike shown in Fig. 3b,g consists of a short-rectangular pulse and a pulse whose amplitude decays exponentially and is thus classified as the RE type. Since the spike shown in Fig. 3c,h consists of two triangle-shaped pulses, it is classified as the TT type. According to such definitions, the spike shown in Fig. 3d,i is classified as the RT type, and the spike shown in Fig. 3e,j is classified as the RR type.

Figures 3p–t present typical STDP curves obtained corresponding to the five different types of superimposed signals (Fig. 3k–o), respectively. In the STDP measurements, we first measured the device conductance *G*_o_ and then applied a superimposed signal. Subsequently, the device conductance *G*_a_ was remeasured. In the STDP curves, we plotted the change in the device conductance Δ*G* (=*G*_o_ − *G*_a_) as a function of Δ*t*. As shown in Fig. 3p–t, the FTJ showed STDP behaviour independently of different superimposed signals, i.e., pre- and post-spikes. However, the shape of STDP curves, which is characterized by the total modulation of the conductivity (Δ*G*_max_) and the time window (*τ*_C_) in this study, depended on the type of pre- and post-spikes, although the peak amplitude |*V*_peak_| of spikes was set to the same value for all measurements.

Note that for the measurements of Fig. 3p–t, the peak amplitude of the pre- and post-spikes was set to 1.25 V. When Δ*t* was much larger than the spike width (≫4 ms), the spikes were not superimposed, and |*V*_FTJ_| remained within 1.25 V. As Δ*t* approached 0, the spikes were superimposed, and |V_FTJ_| exceeded 1.25 V, which was larger than the threshold voltage for RS. As a result, the FTJ showed a non-zero Δ*G* at approximately Δ*t* = 0, but Δ*G* = 0 at Δ*t* = 0.

We also note that the STDP curves shown in Fig. 3 were obtained when the initial states of the device were set to an intermediate conductance state between the lowest and highest conductance states in the HSL. In this condition, the STDP curves were nearly symmetric. However, if the initial states of the device were set to close to the lowest or highest conductance states, decrease or increase in conductance became nearly zero, respectively, resulting in asymmetric STDP curves.

### Dependence of spike types and parameters on the STDP curve

**Figure 4:**
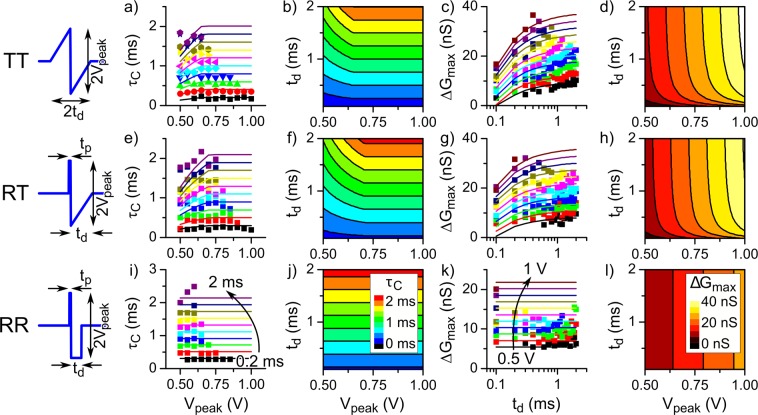
Spike-type dependence of the STDP characteristics. *V*_peak_ dependence of τ_c_ for (**a)** TT-, (**e)** RT-, and (**i)** RR-type spikes. Solid lines in (**a**,**e**,**i**) are calculated results in accordance with Eqs. (2), (5), and (6) with the *V*_th_ value obtained from analysis of Δ*G*_max_, respectively. The *t*_d_ dependence of Δ*G*_max_ for (**c**) TT-, (**g**) RT-, and (**k**) RR-type spikes. Solid lines in (**c**,**g**,**k**) indicate the fitting results in accordance with Eqs. (8)–(10), respectively. Fitting parameters (*V*_th_, Δ*t*, and *C*) are (0.65 ± 0.02 V, 0.079 ± 0.003 ms, and 28.1 ± 0.9 nS/V), (0.71 ± 0.02 V, 0.11 ± 0.01 ms, and 28 ± 0.7 nS/V), and (0.67 V and 16.3 ± 0. 3 nS/V) for TT-, RT-, and RR-type spikes, respectively. Simulated contour plots of τ_C_ for (**b**) TT-, (**f**) RT-, and (**j**) RR-type spikes. Simulated contour plot Δ*G*_max_ for (**d**) TT-, (**h**) RT-, and (**l)** RR-type spikes.

As mentioned above, the STDP characteristics of our FTJ depend on the type of spike. To gain more insight into the STDP characteristics, we evaluated the dependence of the spike types and parameters on the STDP curve. In this study, we chose three types of spikes, i.e., TT, RT, and RR, from the five types. This selection is because the STDP curves obtained using the EE-type and TT-type spikes were almost equivalent, and those obtained using the RE-type and RT-type spikes were almost equivalent, as shown in Fig. 3. We also systematically changed the peak amplitude (*V*_peak_) and the time widths of two pulses constituting spikes. Here, we define two time-parameters: *t*_p_ is the time width of the first rectangular-shaped pulse in the RT- and RR-types of spikes, and *t*_d_ is the time width of the triangle-shaped pulse in the TT- and RT-type spikes and of the second rectangular-shaped pulse in the RR-type spike, as shown in Fig. 4. For each spike type, we performed STDP experiments by changing |*V*_peak_| from 0.5 V to 1 V in steps of 0.05 V and *t*_d_ from 0.1 ms to 2 ms in steps of 0.1 ms. We also used three different *t*_p_ values of 0.1 ms, 0.2 ms and 0.3 ms.

From the STDP curves, we extracted Δ*G*_max_ and τ_C_ (see Methods). Figure 4 shows the *V*_peak_ and *t*_d_ dependences of these two values for the TT-, RT-, and RR-type spikes with *t*_p_ = 0.1 ms. Note that the results for *t*_p_ = 0.2 ms and 0.3 ms are presented in the Supplementary Information. We did not perform the measurements for higher *V*_peak_ values and longer *t*_d_ values in order to avoid the degradation of the devices. In the experiments, the total energy dissipated by the device *E*_max_ was limited to ~5 μJ.

**Figure 5:**
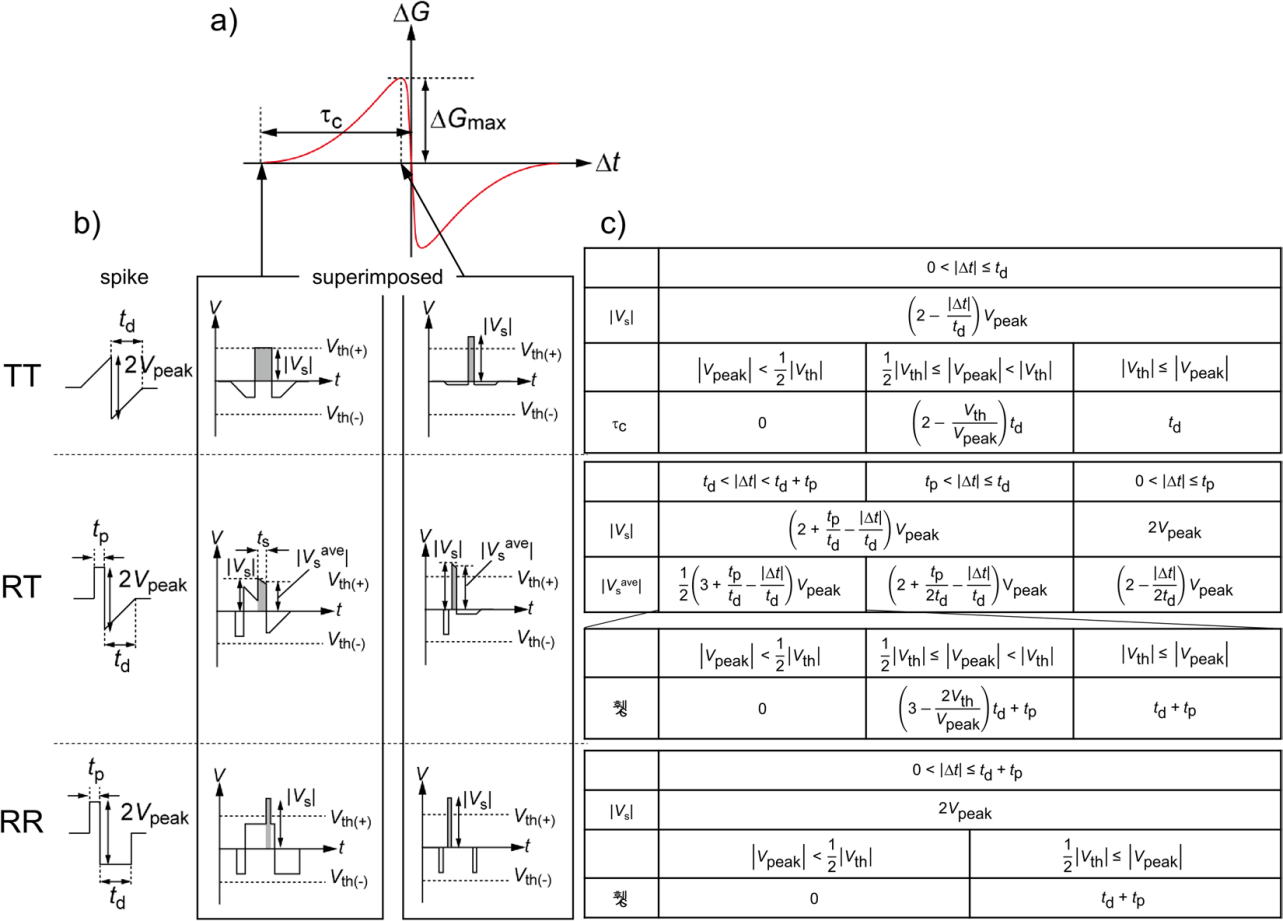
Ideal STDP characteristics. (**a**) Schematic image of the STDP curve. (**b**) Schematic images of the pre-/post-spikes and superimposed spikes at Δ*t* ≈ τ_C_ and Δ*t*, at which Δ*G* ≈ Δ*G*_max_ for three spike types (TT, RT, and RR types). |*V*_peak_| is assumed to be under the condition 1/2|*V*_th_| ≤ |*V*_peak_| < |*V*_th_|. (**c)** Summary of the functions of |*V*_s_|, |*V*_s_^ave^|, and τ_C_ for the three spike types.

Figure 4a–e,i show the *V*_peak_ dependence of τ_C_ for the TT-, RT-, and RR-type spikes, respectively. For the TT- and RT-type spikes, the τ_c_ increased with *V*_peak_ and *t*_d_. On the other hand, for the RR-type spike, the τ_C_ increased with *t*_d_ but was nearly independent of *V*_peak_ at a fixed *t*_d_. These results can be understood from the relationship between the threshold voltage (*V*_th_) for resistive switching and the peak amplitude of superimposed spikes (*V*_s_), as shown in Fig. 5. In an ideal model, the τ_C_ corresponds to the Δ*t* at which Δ*G* starts to have a finite value for Δ*t* < 0 or Δ*G* becomes zero for Δ*t* > 0. At these Δ*t*_s_, |*V*_s_| ≅ |*V*_th_|

For the TT-type spike, the *V*_s_ is given as

1 $${V}_{{\rm{s}}}=(2-\frac{|\Delta t|}{{t}_{{\rm{d}}}}){V}_{{\rm{peak}}},$$

for 0 < |Δ*t*| ≤ *t*_d_. If |*V*_peak_| < 1/2|*V*_th_|, then τ_C_ should be zero because |*V*_s_| is never larger than |*V*_th_|. This means that no STDP behaviour is observed. For 1/2|*V*_th_| ≤ |*V*_peak_| < |*V*_th_|, the Δ*t* (=τ_C_) at |*V*_s_| ≅ |*V*_th_| is given as

2 $${\tau }_{C}\cong (2-\frac{{V}_{{\rm{t}}{\rm{h}}}}{{V}_{{\rm{p}}{\rm{e}}{\rm{a}}{\rm{k}}}}){t}_{{\rm{d}}}.$$

For |*V*_th_| ≤ |*V*_peak_|, τ_C_ should be *t*_d_ because |*V*_s_| is larger than |*V*_th_| independently of *V*_peak_.

For the RT-type spike, the *V*_s_ is given as

3 $${V}_{{\rm{s}}}=(2+\frac{{t}_{{\rm{p}}}}{{t}_{{\rm{d}}}}-\frac{|\varDelta t|}{{t}_{{\rm{d}}}}){V}_{{\rm{peak}}}.$$

for *t*_p_ < |Δ*t*| ≤ *t*_d_ + *t*_p_. As shown in Fig. 5, however, the spike peak is not rectangular but sawtooth shaped. For the RS of the FTJs, not a peak amplitude of the voltage pulse but a time integral of applied voltage or displacement current is a main determinant of the magnitude of resistance change. This is because a ratio between the switched and unswitched ferroelectric domains in a ferroelectric barrier is proportional to the charges accumulated in the metal electrodes. For the sawtooth-shaped spike, therefore, the average amplitude (*V*_s_^ave^) seems to be a good feature quantity of the spike peak since a time integral of the applied voltage corresponds to *V*_s_^ave^ × *t*_s_, where *t*_s_ is the time duration of the spike peak (Fig. 5b). The *V*_s_^ave^ is given as

4 $${V}_{{\rm{s}}}^{{\rm{ave}}}=\frac{1}{2}(3+\frac{{t}_{{\rm{p}}}}{{t}_{{\rm{d}}}}-\frac{|\Delta t|}{{t}_{{\rm{d}}}}){V}_{{\rm{peak}}}.$$

If |*V*_peak_| < 1/2|*V*_th_|, then τ_C_ should be zero. For 1/2|*V*_th_| ≤ |*V*_peak_| < |*V*_th_|, the τ_C_ at |*V*_s_^ave^| ≅ |*V*_th_| is given as

5 $${\tau }_{C}\cong (3-\frac{2{V}_{{\rm{t}}{\rm{h}}}}{{V}_{{\rm{p}}{\rm{e}}{\rm{a}}{\rm{k}}}}){t}_{{\rm{d}}}+{t}_{{\rm{p}}}.$$

For |*V*_th_| ≤ |*V*_peak_|, τ_C_ should be *t*_d_ + *t*_p_ independently of *V*_peak_.

Equations (2) and (5) mean that the τ_C_ increases with *V*_peak_ and *t*_d_. These results seem to be qualitatively consistent with the results of Fig. 4a,e if 1/2|*V*_th_| ≤ |*V*_peak_| < |*V*_th_| for these experiments. On the other hand, for the RR-type spike, *V*_s_ = 2*V*_peak_ (=constant), when Δ*t* < *t*_d_ + *t*_p_ and 1/2|*V*_th_| ≤ |*V*_peak_|. Therefore, the τ_C_ is given as

6 $${\tau }_{C}={t}_{{\rm{d}}}+{t}_{{\rm{p}}},$$

independently of *V*_peak_. This finding seems to also be qualitatively consistent with the result of Fig. 4i, if 1/2|*V*_th_| ≤ |*V*_peak_| for this experiment.

Figures 4c,g,k show the *t*_d_ dependence of Δ*G*_max_ for the TT-, RT-, and RR-type spikes, respectively. For the TT- and RT-type spikes, Δ*G*_max_ increased with *t*_d_ and *V*_peak_, while Δ*G*_max_ was nearly independent of *t*_d_ at a fixed *V*_peak_ for the RR-type spike. These results can be understood qualitatively from the *t*_d_ dependence of *V*_S_ or *V*_s_^ave^. Here, we assume that the Δ*G*_max_ is given as a monotonically increasing function of (*V*_S_ − *V*_th_); Δ*G*_max_ = *f*(*V*_S_ − *V*_th_). According to Eq. (1), *V*_s_ increases with *V*_peak_ and *t*_d_ for the TT-type spike. As a result, Δ*G*_max_ increases with *V*_peak_ and *t*_d_. In the RT-type spike, *V*_s_ is 2*V*_peak_ for 0 < |Δ*t*| ≤ *t*_p_, independently of *t*_d_. As discussed above, however, *V*_s_^ave^ seems to be a good feature quantity of the spike peak for the RT-type spike, and the *V*_s_^ave^ is given as

7 $${V}_{{\rm{s}}}^{{\rm{ave}}}=(2-\frac{|\Delta t|}{2{t}_{{\rm{d}}}}){V}_{{\rm{peak}}},$$

meaning that *V*_s_^ave^ increased with *V*_peak_ and *t*_d_. Therefore, Δ*G*_max_ is expected to increase with *V*_peak_ and *t*_d_ for the RT-type spike. In contrast to the TT- and RT-type spikes, *V*_s_ is 2*V*_peak_ (constant) independently t_d_ for the RR-type spike. Therefore, Δ*G*_max_ for the RR-type spike is constant independently of *t*_d_ at a fixed *V*_peak_.

### Analysis of τ_C_ and ΔG_max_

Here, we analyse the experimental results based on the above-mentioned equations. To quantitatively analyse the Δ*G*_max_, the function Δ*G*_max_ = *G*[*V*_spike_(*t*)] must be determined, where *G*[*V*_spike_(*t*)] may be an integral function and *V*_spike_(*t*) is the time evolution of spike amplitude. However, it is difficult to determine *G*[*V*_spike_(*t*)]. We thus introduced empirical equations: (*V*_s_ − *V*_th_) or (*V*_s_^ave^ − *V*_th_) instead of *V*_spike_(*t*) and introduced the function Δ*G*_max_ = *f*(*V*_S_ − *V*_th_) or *f*(*V*_s_^ave^ − *V*_th_). Because our FTJs showed the threshold-like HSL (Fig. 2c), we assume that the *f*(*V*_S_ − *V*_th_) or *f*(*V*_s_^ave^ − *V*_th_) are threshold functions as

8 $$\begin{array}{rcl}\Delta {G}_{{\rm{\max }}} & = & \{\begin{array}{cc}0 & ;\,{V}_{{\rm{s}}} < {V}_{{\rm{th}}}\\ C\,({V}_{{\rm{s}}}-{V}_{{\rm{th}}}) & ;\,{V}_{{\rm{s}}} > {V}_{{\rm{th}}}\end{array}\\ & = & \{\begin{array}{cc}0 & ;\,(2-\frac{|\Delta t|}{{t}_{{\rm{d}}}}){V}_{{\rm{peak}}} < {V}_{{\rm{th}}}\\ C[(2-\frac{|\Delta t|}{{t}_{{\rm{d}}}}){V}_{{\rm{peak}}}-{V}_{{\rm{th}}}] & ;\,(2-\frac{|\Delta t|}{{t}_{{\rm{d}}}}){V}_{{\rm{peak}}} > {V}_{{\rm{th}}}\end{array}\end{array},$$

9 $$\begin{array}{ccc}\Delta {G}_{max} & = & \{\begin{array}{cc}0 & ;\,{V}_{{\rm{s}}}^{{\rm{a}}{\rm{v}}{\rm{e}}} < {V}_{{\rm{t}}{\rm{h}}}\\ C\,({V}_{{\rm{s}}}^{{\rm{a}}{\rm{v}}{\rm{e}}}-{V}_{{\rm{t}}{\rm{h}}}) & ;\,{V}_{{\rm{s}}}^{{\rm{a}}{\rm{v}}{\rm{e}}} > {V}_{{\rm{t}}{\rm{h}}}\end{array}\\ & = & \{\begin{array}{cc}0 & ;\,(2-\frac{|\Delta t|}{2{t}_{{\rm{d}}}}){V}_{{\rm{p}}{\rm{e}}{\rm{a}}{\rm{k}}} < {V}_{{\rm{t}}{\rm{h}}}\\ C[(2-\frac{|\varDelta t|}{2{t}_{{\rm{d}}}}){V}_{{\rm{p}}{\rm{e}}{\rm{a}}{\rm{k}}}-{V}_{{\rm{t}}{\rm{h}}}] & ;\,(2-\frac{|\Delta t|}{2{t}_{{\rm{d}}}}){V}_{{\rm{p}}{\rm{e}}{\rm{a}}{\rm{k}}} > {V}_{{\rm{t}}{\rm{h}}}\end{array}\end{array},$$

10 $$\begin{array}{ccc}\Delta {G}_{max} & = & \{\begin{array}{cc}0 & ;\,{V}_{{\rm{s}}} < {V}_{{\rm{t}}{\rm{h}}}\\ C\,({V}_{{\rm{s}}}-{V}_{{\rm{t}}{\rm{h}}}) & ;\,{V}_{{\rm{s}}} > {V}_{{\rm{t}}{\rm{h}}}\end{array}\\ & = & \{\begin{array}{cc}0 & ;\,2{V}_{{\rm{p}}{\rm{e}}{\rm{a}}{\rm{k}}} < {V}_{{\rm{t}}{\rm{h}}}\\ C(2{V}_{{\rm{p}}{\rm{e}}{\rm{a}}{\rm{k}}}-{V}_{{\rm{t}}{\rm{h}}}) & ;\,2{V}_{{\rm{p}}{\rm{e}}{\rm{a}}{\rm{k}}} > {V}_{{\rm{t}}{\rm{h}}}\end{array}\end{array},$$

for the TT-, RT-, and RR-type spikes, respectively. To analyse the results using these equations, the *V*_th_ should be determined. In an actual FTJ, however, it is difficult to definitely determine the *V*_th_ because the HSL did not show a clear threshold behaviour (Fig. 2c). Therefore, to obtain a *V*_th_ value, we first fitted the results for the TT-type and the RT-type spikes by Eqs. (8) and (9), respectively. The solid lines in Fig. 4c,g indicate that the fitting results are in accordance with Eqs. (8) and (9), respectively and are in fairly good agreement with the experimental data. From the fitting results, we obtained a *V*_th_ value of 0.63–0.73 V. Note that the prefactors of *C* were also obtained from the fitting results. Using the averaged *V*_th_ value of 0.67 V, we then fitted the results for the RR-type spikes by Eq. (10), as indicated by the solid lines in Fig. 4k. Note that the values of fitting parameters (*V*_th_, Δ*t*, and *C*) are described in the figure caption.

Next, τ_C_ for the TT-, RT- and RR-type spikes are analysed using Eqs. (2), (5) and (6), respectively. Here, we used the averaged *V*_th_ value obtained from the analysis of Δ*G*_max_. The solid lines in Fig. 4a,e,i are the calculated results of Eqs. (2), (5) and (6), respectively and are in fairly good agreement with the experimental data. These results confirm the validity of the equations for the analysis of the experimental results.

### Contour plots of τ_C_ and ΔG_max_

**Figure 6:**
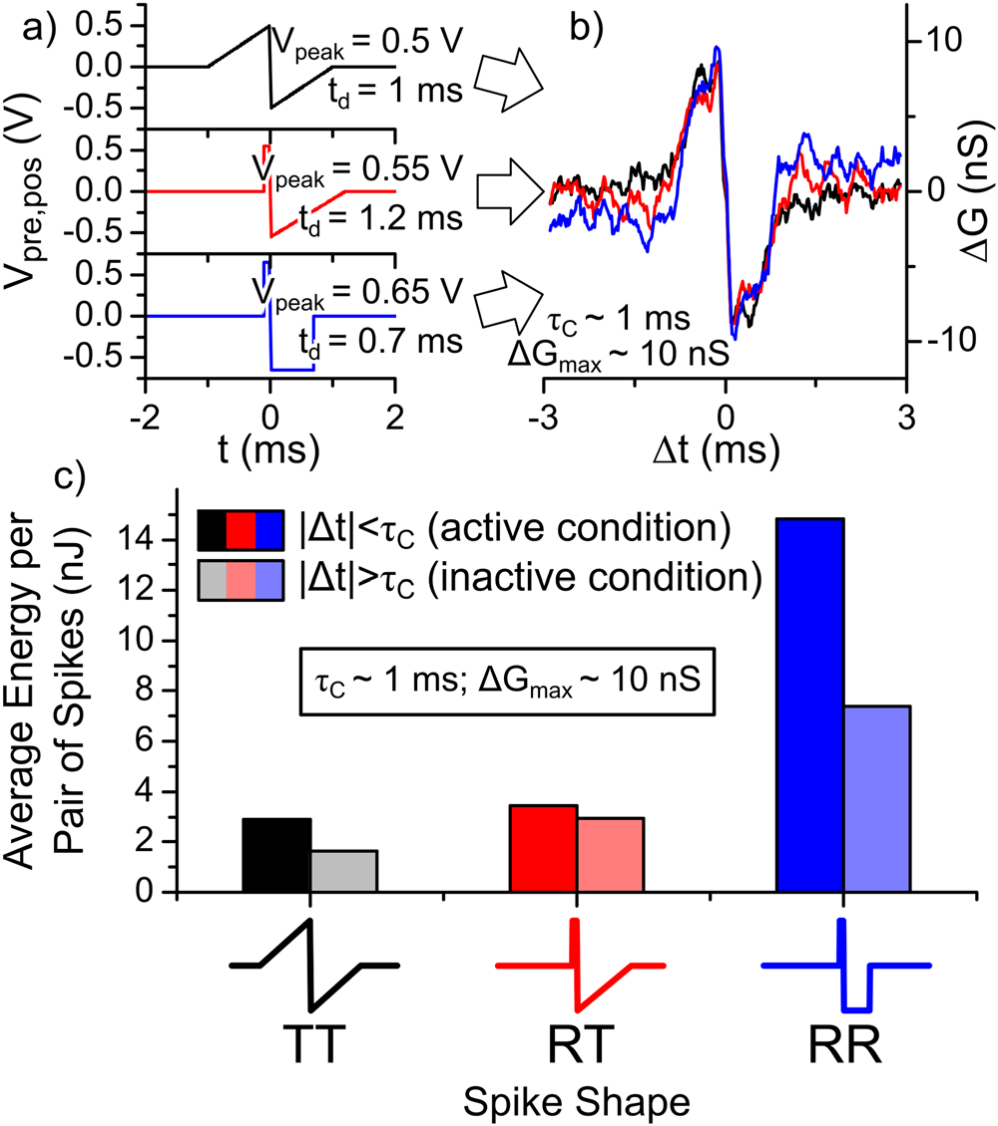
STDP curves and power consumption of the artificial synapses implemented by FTJ. (**a**) TT-type (black), RT-type (red), and RR-type (blue) spikes, and (**b**) respective STDP curves. (**c**) Averaged energy dissipated in the FTJ for the three types of spikes. See text for details.

We can simulate contour plots of τ_C_ and Δ*G*_max_ as a function of *t*_d_ and *V*_peak_ by using the above-mentioned equations with parameters obtained from analyses of the experimental results. Figures 4d,h,l are the contour plots of Δ*G*_max_ and Fig. 4b,f,j are those of τ_C_ for the TT-, RT-, and RR-type spikes, respectively. These contour plots can serve as a guide for controlling the STDP characteristics. This means that we can obtain a STDP curve with the desired τ_C_ and Δ*G*_max_ values by choosing the appropriate *t*_d_ and *V*_peak_ values, using these contour plots as a guideline. As shown in Fig. 6a, for instance, the STDP curves with τ_C_ = 1 ms and ΔG_max_ = 10 nS were obtained for the TT-, RT-, and RR-type spikes. The *t*_d_ and *V*_peak_ values used in these demonstrations were 1 ms and 0.5 V for the TT type, 1.2 ms and 0.55 V for the RT type, and 0.7 ms and 0.65 V for the RR type.

### Measurements of the power consumption

One of the advantages of the SNN is low power consumption. As shown in Fig. 6, although the STDP characteristics are similar, the spike parameters used for obtaining the STDP curves are different among the spike types. This difference suggests that the power consumption depends on the spike types. To evaluate the spike type dependence of the power consumption, we measured the energy dissipated in the FTJ by conducting a sequence of 1000 pairs of pre- and post-spikes with randomly distributed Δ*t* between −3 ms and 3 ms for the TT-, RT-, and RR-type spikes. The spike parameters were the same as in the experiments of Fig. 6a. We divided the data into two groups depending on whether |Δ*t*| is larger than or smaller than τ_C_ (=1 ms). Figure 6b presents the average energy per pair of pre- and post-spikes for each type of spike. The results show that the power consumption for the TT-type spike was smallest among the three spike types and that the power consumption for the RR-type spike was 3–5 times larger than that for the TT- and RT-type spikes. The energy dissipated in the FTJ is given as a time integral of *I*(*t*)*V*(*t*). If the FTJ shows a linear *I*-*V* curve because the dissipated energy is proportional to a time integral of *V*(*t*)^2^, then the power consumption for the RT-type spike is smallest among the three spike types. This discrepancy seems to be due to the nonlinearity of the *I*-*V* curve of the FTJ (Fig. 1b). In this case, the dissipated energy nonlinearly increases with voltage. Because the *V*_peak_ value and the resultant *V*_s_ value of the RT-type spike were larger than those of the TT-type spike, the dissipated energy for the RT-type spike might be larger than that for the TT-type spike in this study. For the RR-type spike, since rectangular pulses (i.e., constant voltage pulses) were applied to the FTJ, its power consumption was larger than that for the TT- and RT-type spikes.

## Discussion

Here, we discuss which spike type is better for implementing an SNN with FTJs showing nonlinear *I*-*V* curves and HSLs, similar to our FTJs. From an engineering point of view, the RR-type spike is better because it can be generated by a simple electronic circuit and thus presumably uses less die area. As shown in Fig. 6b, however, its power consumption was 3–5 times larger than that for the TT- and RT-type spikes. On the other hand, the TT-type spike seems to be better at implementing an SNN in terms of power consumption. Nevertheless, its implementation is not adequate in real neuromorphic systems, unless a long delay time (*t*_d_) with a triangular onset is utilized^24^. In our FTJ, the power consumption for the RT-type spike was slightly larger than that for the TT-type spike. As mentioned above, however, the power consumption depends on the nonlinearity of the *I*-*V* curve of the device. If the nonlinearity of the *I*-*V* curve can be optimized to reduce the power consumption, then the RT-type spike will be adequate to implement an SNN in terms of the power consumption and operating time.

It is well known that STDP is based on the Hebbian concept, namely, synaptic connections between neurons that fire together are reinforced^7^. However, synapses connecting uncorrelated neurons never become reinforced. Therefore, only a few active synapses are often reinforced by winner-takes-all algorithms^25^. In general, one neuron has thousands of synaptic connections, but only a few of them, i.e., active synapses, contribute to the computation. The active synapses that have relatively high conductivity transmit pre- and post-spikes with |Δ*t*| < τ_C_. In an SNN circuit, however, inactive synapses also receive pre- and post-spikes with |Δ*t*| > τ_C_ and consume energy. Since the number of inactive synapses is larger than that of active synapses in a general SNN circuit, the total power consumption in inactive synapses is not negligible. As shown in Fig. 6b, the averaged power consumption in the inactive FTJs was 50–82% of that in active FTJs. If an SNN circuit is fabricated using our FTJs, then inactive synapses will be responsible for most of the power consumption. The power consumption of a FTJ under inactive-synapse condition depends on its resistance in the lower voltage range (<|*V*_th_|). Therefore, controlling the nonlinearity of the *I*-*V* curve, i.e., increasing the resistance in the lower voltage range (<|*V*_th_|), can reduce the power consumption in inactive synapses.

## Conclusions

We investigated the spike-type dependence of STDP characteristics for BaTiO_3_-based FTJs. To analyse the STDP curves characterized by an amplitude of the conductivity modulation (ΔG_max_) and a time window (τ_C_), we proposed empirical equations for three different spike types (triangle-triangle, rectangular-triangle, and rectangular-rectangular spikes) by taking into account the time evolution of the peak amplitude (*V*_s_) of superimposed voltage spikes, the relationship between *V*_s_ and the threshold voltage (*V*_th_) for resistive switching, and the nonlinearity of the *I*-*V* curve of the FTJs. The proposed equations could reproduce the experimental results and provide a guideline for controlling the STDP characteristics. From the power consumption experiments of STDP, we found that the power consumption under the inactive-synapse condition (|Δ*t*| > τ_C_) is too large to ignore when an SNN is implemented by using our FTJs as synapses. Because the power consumption of a FTJ under inactive-synapse condition depends on its resistance in the lower voltage range (<|*V*_th_|), increase in the resistance at <|*V*_th_| is one of the solutions for this issue. The power consumption under the inactive-synapse condition may be an issue for an SNN circuit consisting of other FTJs, because the nonlinearity of *I-V* characteristics of other FTJs are similar to our FTJs. Therefore, this study will give a guideline for investigations of power consumption in neural network circuits consisting of FTJs.

## Methods

### Fabrication of FTJs

Oxide films were fabricated by pulsed-laser deposition using a KrF excimer laser. An SrRuO_3_ (SRO) bottom-electrode layer was grown on a DyScO_3_ substrate at a substrate temperature (T_sub_) of 600 °C and an oxygen pressure (P_O2_) of 0.1 Pa. A BTO barrier layer was subsequently deposited at T_sub_ ≈ 650 °C and P_O2_ ≈ 4.5 Pa. After the depositions, the heterostructures were cooled slowly in an atmosphere of oxygen (P_O2_ ≈ 10 Pa). The thicknesses of the SRO and BTO layers were 30 nm and 3.2 nm (8-unit cells), respectively. A 10-nm-thick Pt top-electrode layer was fabricated by electron-beam (EB) deposition. Subsequently, a cover-layer of Au (20 nm) was fabricated by EB deposition. The Au/Pt/BTO/SRO layered structure was patterned into 2 × 2 μm^2^ junctions by conventional photolithography and Ar ion milling. For an insulation between the top and bottom electrodes, a SiO_2_ layer (300 nm) was deposited by sputtering and patterned by a self-aligned lift-off method. Finally, an Au(400 nm)/Ti(10 nm) wire metal for the top electrode was fabricated by EB deposition and the lift-off method.

### Electrical characterizations of the FTJs

To characterize the FTJs, we developed a homemade source-measuring unit (SMU) and an associated acquisition software based on LabVIEW (National Instruments). This system can apply arbitrary voltage waveforms to devices and measure the current with sampling rates up to 625 kS/s. The devices were set in a Karl Suss PM8 Probe Station. For the *I*-*V* and HSL measurements, the writing voltage pulse lasted for 3 ms, and the device current was acquired with a noise level ~1 µA at a sampling rate of 10 kS/s (bandwidth = 40 kHz). We discarded the first ten measurements and averaged the remaining 20 measurements to obtain *I*_pulse_ (Fig. 2b). For the HSL measurements, the reading voltage of 0.25 V with a time duration of 20 ms was applied after each writing pulse, and the current was acquired with a noise level <1 nA at a rate of 10 kS/s (bandwidth = 600 Hz). We discarded the first 50 measurements and averaged the remaining 150 measurements to obtain a conductance *G* in the HSL (Fig. 2c).

### Measurement and parameter extraction from the STDP curves

We used the same setup for the *I*-*V* and HSL measurements to measure the STDP curves. We generated a long waveform (80000 samples in 8 s) containing 80 sets of pre- and post-spikes; each one occupies a time slot of 95 ms. After applying a pair of pre- and post-spikes, we applied a reading voltage of 0.25 V with a time duration of 5 ms to measure the conductance of the device. The first pair of pre- and post-spikes had Δ*t* = 8 ms. The second one had Δ*t* = −8 ms (i.e., post before pre). After the second one, pairs of pre- and post-spikes were applied by alternating positive and negative Δ*t* and by decreasing |Δ*t*| to 0 s in steps of 0.2 ms. After the measurements, a STDP curve was constructed by calculating the conductivity change (Δ*G*) between consecutive measurements. Note that experiments for shorter spike duration (<0.1 ms) were not conducted in this study because of limits in our experimental set-up (noise, speed).

To extract the amplitude of the conductivity modulation (Δ*G*_max_), the time window (τ_C_), and the difference of Δ*G* between Δ*t* < 0 and Δ*t* > 0 (ΔG_0_), we multiplied the Δ*G* of STDP curve by −1 for Δ*t* > 0 and obtained a modified STDP curve. The modified STDP curves were fitted with the formula Δ*G* = a/(1 + exp ((Δt − b)/c)). Then, Δ*G*_max_ and τ_C_ were extracted as Δ*G*_max_ = a and τ_C_ = −(c × ln(1/p − 1) + b), where ln(*x*) is the natural logarithm. The parameter p was chosen between 0.02–0.33 for each dataset to have a proper matching with Eqs. (2), (5) and (6).

## Supplementary information

Supplementary Information
