# Supplementary material for: Spike-shape dependence of the spike-timing dependent synaptic plasticity in ferroelectric-tunnel-junction synapses
Source: Sci Rep. 2019 Nov 28;9:17740. doi: 10.1038/s41598-019-54215-w (PMC6882828; doi:10.1038/s41598-019-54215-w)
Supplement: Supplementary file 1 — Supplementary Information [file 41598_2019_54215_MOESM1_ESM.docx]

Spike-shape dependence of the spike-timing dependent synaptic plasticity in ferroelectric-tunnel-junction synapses
- Supplementary Information -

*P. Stoliar*, H. Yamada, Y. Toyosaki, and A. Sawa*

National Institute of Advanced Industrial Science and Technology (AIST),
AIST Tsukuba Central 5, 1-1-1 Higashi, Tsukuba, Ibaraki 305-8565, Japan
E-mail: [p.stoliar@aist.go.jp](mailto:p.stoliar@aist.go.jp)


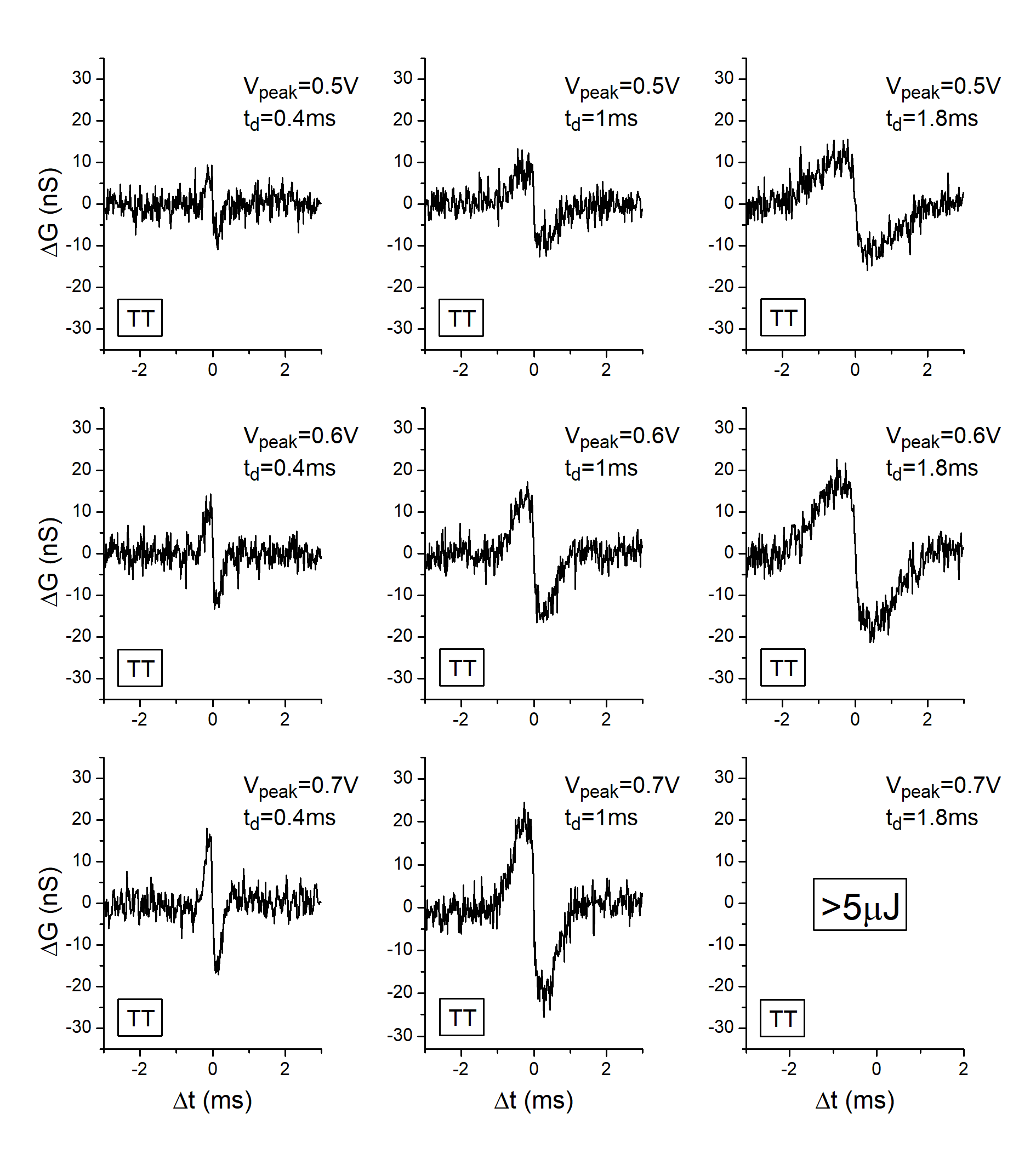


**Figure S1.** Typical STDP curves for TT-type spikes with different *V*_peak_ and *t*_d_. They correspond to 8 datapoints in Figs. 4a and 4c.

**
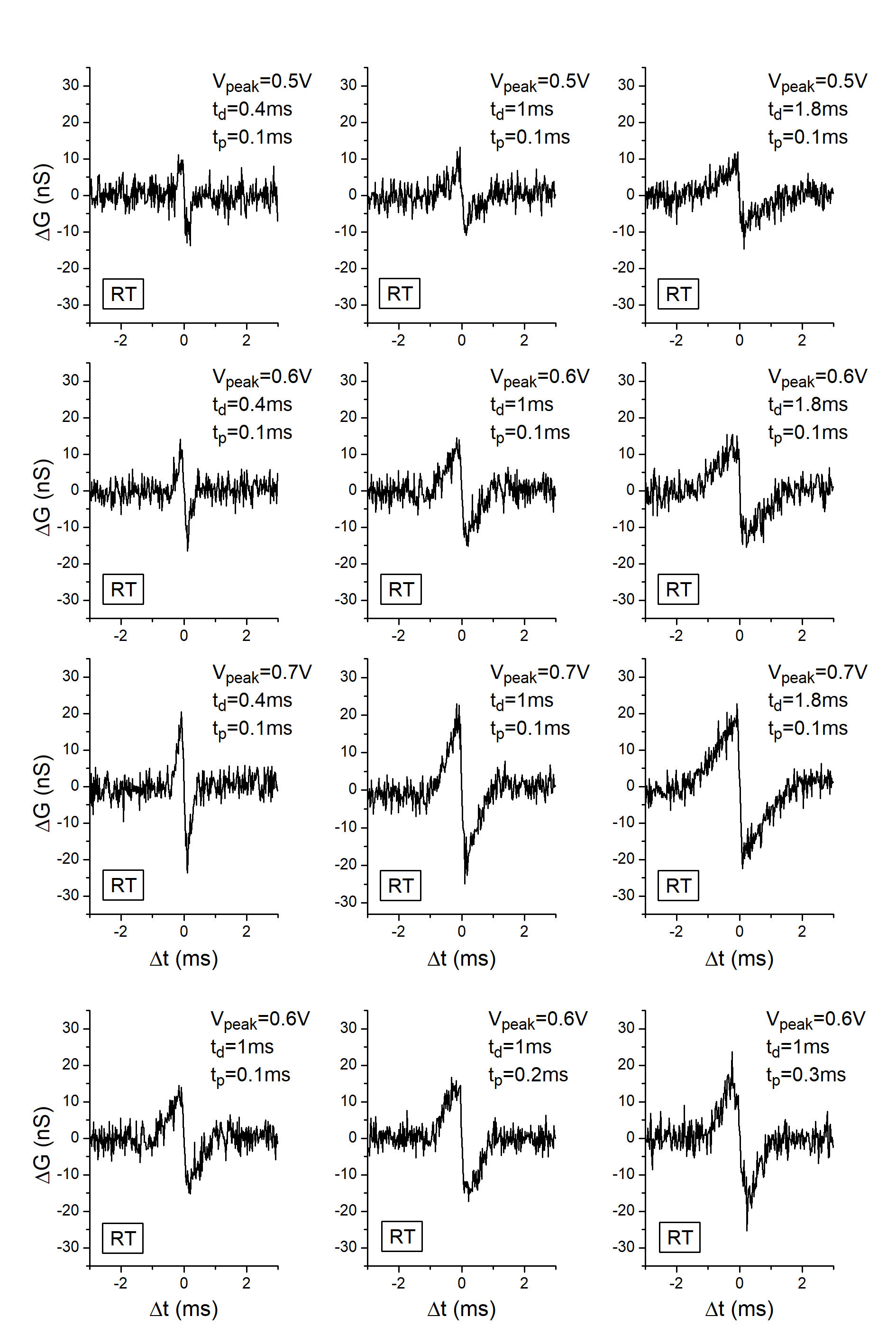
**

**Figure S2.** Typical STDP curves for RT-type spikes with different *V*_peak_, *t*_d_, and *t*_p_. They correspond to 9 datapoints in Figs. 4e and 4g.

**
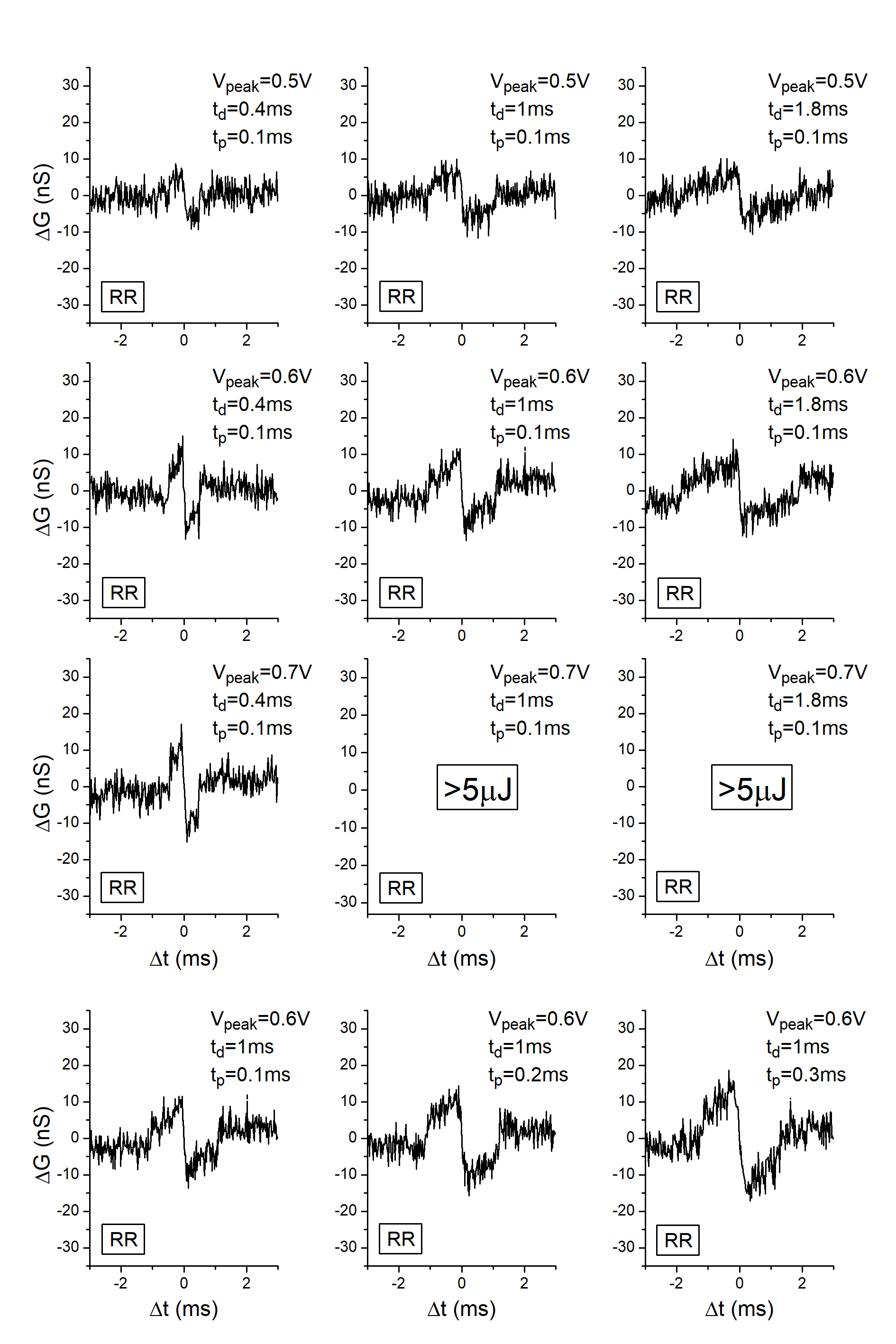
**

**Figure S3.** Typical STDP curves for RR-type spikes with different *V*_peak_, *t*_d_, and *t*_p_. They correspond to 7 datapoints in Figs. 4i and 4k.

| **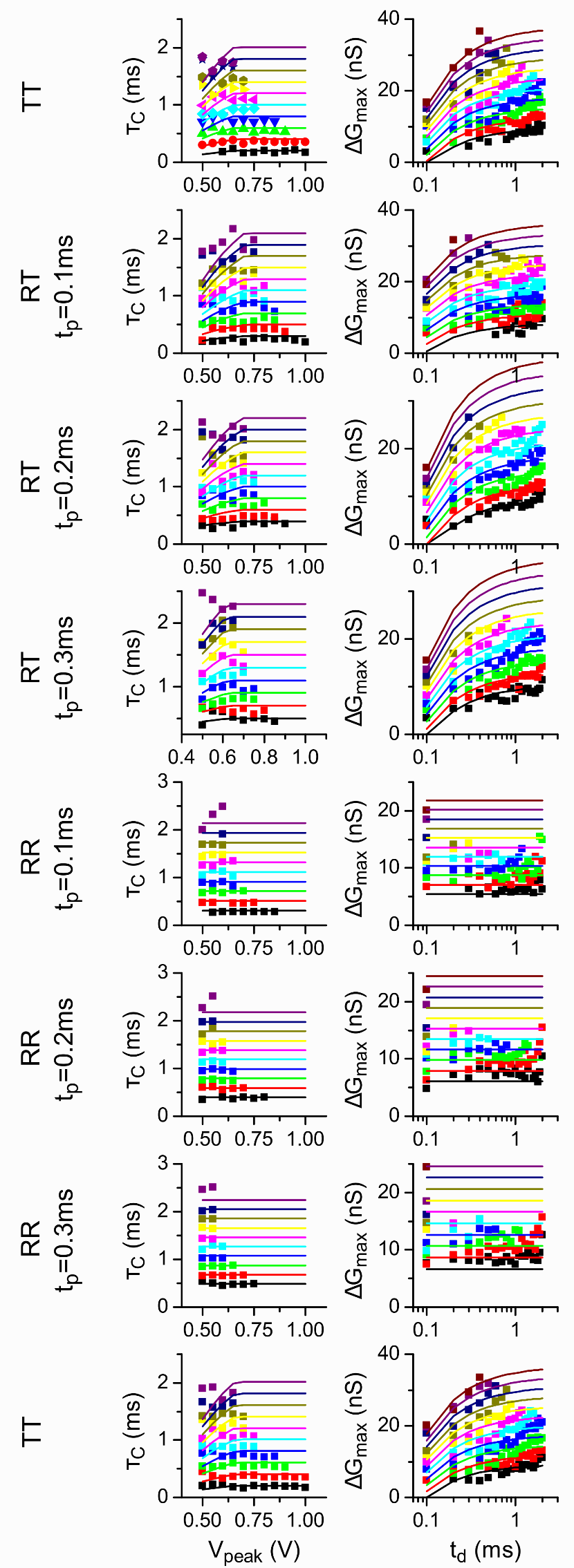** | **Figure S4.** *V*_peak_ dependence of τ_c_ and *t*_d_ dependence of Δ*G*_max_ for the TT-, RT-, and RR-type spikes. The results are longitudinally arranged in the order that the measurements were done. Note that the measurements for the TT-type spikes were carried out twice: the second measurement was carried out at the end of series of measurement in order to confirm the reproducibility of measured results. The result of the second measurement (bottom) is equivalent for that of the first measurement (top), confirming that the degradation of the device during the experiments was negligible. |
| --- | --- |

**
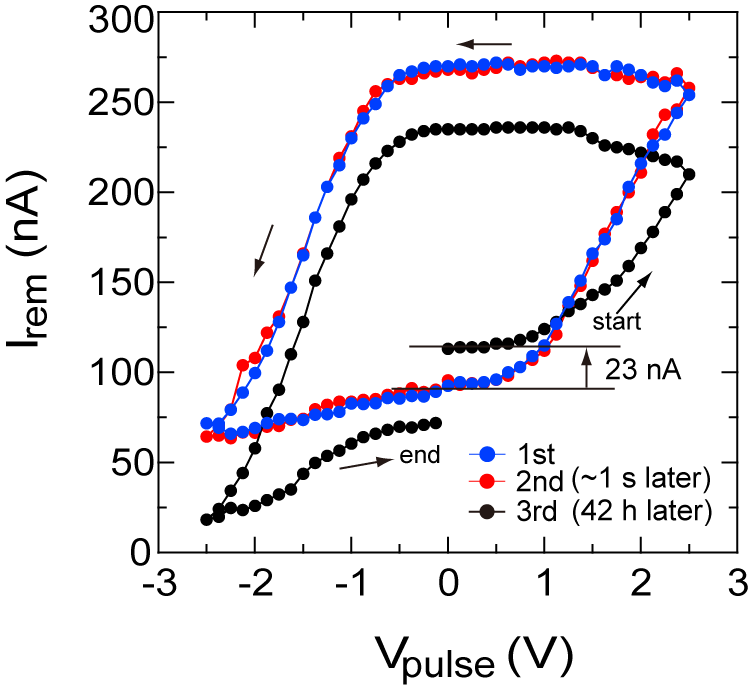
**

**Figure S4.** Hysteresis switching loops (HSLs) of the FTJ measured by applying pulsed voltages with a pulse duration of 3 ms. The second measurement (red dots) was carried out after about 1 second of the first measurement (blue dots). These HSLs confirms a reproducibility of HLS measurements. The third measurement (black dots) was carried out after about 42 hours of the second measurement. The remnant current (I_rem_) at the high resistance state (HRS) increased from 90 nA to 113 nA after 42 hours. The rate of I_rem_ change was about 0.6%/hour, confirming that the FTJ shows practically nonvolatile resistive switching sufficient for STDP experiments.
